# Recent Advances in the Pathophysiology of Musculocontractural Ehlers-Danlos Syndrome

**DOI:** 10.3390/genes11010043

**Published:** 2019-12-29

**Authors:** Tomoki Kosho, Shuji Mizumoto, Takafumi Watanabe, Takahiro Yoshizawa, Noriko Miyake, Shuhei Yamada

**Affiliations:** 1 Department of Medical Genetics, Shinshu University School of Medicine, Matsumoto 390-8621, Japan; 2 Center for Medical Genetics, Shinshu University Hospital, Matsumoto 390-8621, Japan; 3 Research Center for Supports to Advanced Science, Matsumoto 390-8621, Japan; 4 Department of Pathobiochemistry, Faculty of Pharmacy, Meijo University, Nagoya 468-8503, Japan; mizumoto@meijo-u.ac.jp (S.M.); shuheiy@meijo-u.ac.jp (S.Y.); 5 Laboratory of Anatomy, School of Veterinary Medicine, Rakuno Gakuen University, Ebetsu 069-8501, Japan; t-watanabe@rakuno.ac.jp; 6 Division of Animal Research, Research Center for Supports to Advanced Science, Shinshu University, Matsumoto 390-8621, Japan; tyoshizawa@shinshu-u.ac.jp; 7 Department of Human Genetics, Yokohama City University Graduate School of Medicine, Yokohama 236-0004, Japan; nmiyake@yokohama-cu.ac.jp

**Keywords:** musculocontractural Ehlers–Danlos Syndome, carbohydrate sulfotransferase-14 (CHST14)/dermatan 4-*O*-sulfotransferase-1 (D4ST1), *CHST14*, dermatan sulfate epimerase (DSE), *DSE*, dermatan sulfate (DS), decorin, collagen

## Abstract

Musculocontractural Ehlers–Danlos Syndome (mcEDS) is a type of EDS caused by biallelic pathogenic variants in the gene for carbohydrate sulfotransferase 14/dermatan 4-*O*-sulfotransferase 1 (*CHST14*/*D4ST1*, mcEDS-*CHST14*), or in the gene for dermatan sulfate epimerase (*DSE*, mcEDS-*DSE*). Thus far, 41 patients from 28 families with mcEDS-*CHST14* and five patients from four families with mcEDS-*DSE* have been described in the literature. Clinical features comprise multisystem congenital malformations and progressive connective tissue fragility-related manifestations. This review outlines recent advances in understanding the pathophysiology of mcEDS. Pathogenic variants in *CHST14* or *DSE* lead to reduced activities of relevant enzymes, resulting in a negligible amount of dermatan sulfate (DS) and an excessive amount of chondroitin sulfate. Connective tissue fragility is presumably attributable to a compositional change in the glycosaminoglycan chains of decorin, a major DS-proteoglycan in the skin that contributes to collagen fibril assembly. Collagen fibrils in affected skin are dispersed in the papillary to reticular dermis, whereas those in normal skin are regularly and tightly assembled. Glycosaminoglycan chains are linear in affected skin, stretching from the outer surface of collagen fibrils to adjacent fibrils; glycosaminoglycan chains are curved in normal skin, maintaining close contact with attached collagen fibrils. Homozygous (*Chst14*^−/−^) mice have been shown perinatal lethality, shorter fetal length and vessel-related placental abnormalities. Milder phenotypes in mcEDS-*DSE* might be related to a smaller fraction of decorin DS, potentially through residual DSE activity or compensation by DSE2 activity. These findings suggest critical roles of DS and DS-proteoglycans in the multisystem development and maintenance of connective tissues, and provide fundamental evidence to support future etiology-based therapies.

## 1. Introduction

**Figure 1:**
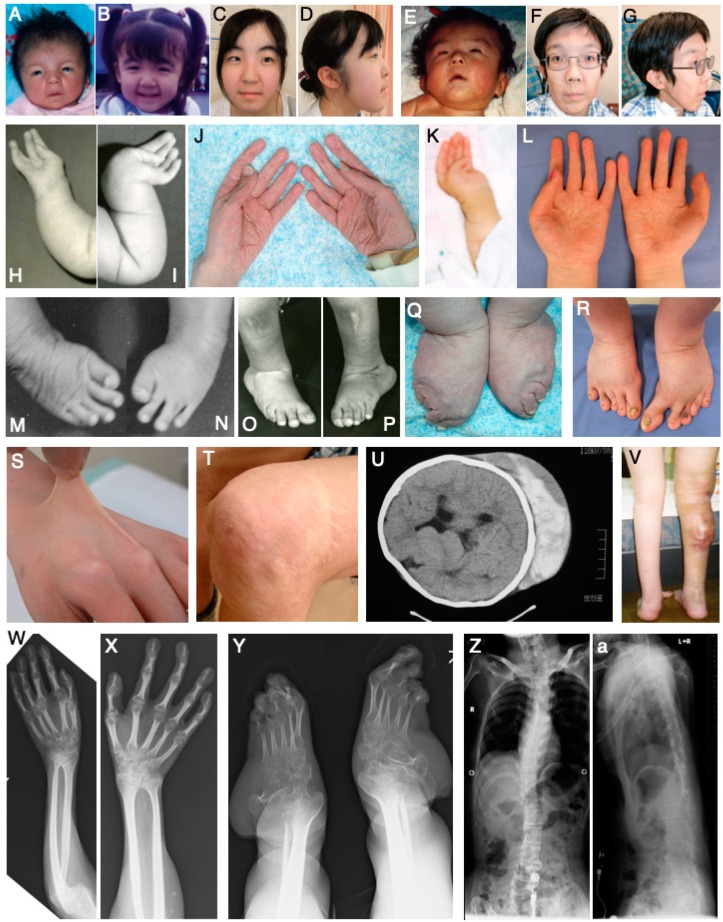
Clinical photographs and radiological images of patients with mcEDS-*CHST14*. Clinical photographs of a patient with heterozygous variants Pro281Leu/Try293Cys at age 23 days (**A**), 3 years (**B**), and 16 years (**C**,**D**); those of a patient with a homozygous variant Pro281Leu at age 2 months (**H**,**M**,**N**), 3 months (**I**), 6 years (**O**,**P**), and 28 years (**J**,**Q**); photographs of a patient with a homozygous variant “P281L” in the neonatal period (**E**) and at age 30 years (**F**,**G**,**T**); and photographs of a patient with heterozygous variants Pro281Leu /Cys289Ser at age 1 month (**K**), 16 years (**V**), and 19 years (**L**,**R**,**S**) [5]. Radiological image of a patient with heterozygous variants Pro281Leu /Try293Cys at age 6 years (**U**); images of a patient with a homozygous variant Pro281Leu at age 28 years (**W**–**Z**,**a**) [5,13]. (U, reproduced from Kosho et al. *Am. J. Med. Genet. Part A* **2005**, *138A*, 282–287, with permission from Wiley-Liss, Inc.; the other images, reproduced from Kosho et al. *Am. J. Med. Genet. Part A* **2010**, *152A*, 1333–1346, with permission from Wiley-Liss, Inc.).

Musculocontractural Ehlers–Danlos Syndome (mcEDS) is a type of EDS, caused by biallelic pathogenic variants in the gene for carbohydrate sulfotransferase 14/dermatan 4-*O*-sulfotransferase 1 (*CHST14*/*D4ST1*, mcEDS-*CHST14*) (MIM#601776), or in the gene for dermatan sulfate epimerase (*DSE*, mcEDS-*DSE*) (MIM#615539) [1,2,3]. mcEDS-*CHST14* was originally described as three independent conditions: A rare type of arthrogryposis syndrome “adducted thumb-clubfoot syndrome” [4]; a specific type of EDS “EDS, Kosho type” [5,6]; and a subset of kyphoscoliosis type without lysyl hydroxylase deficiency [7]. To date, 41 patients from 28 families have been reported to have mcEDS-*CHST14* [4,5,6,7,8,9,10,11,12,13,14,15,16,17,18,19,20,21,22]. mcEDS-*DSE* was identified in a patient with a phenotype similar to that of patients with mcEDS-*CHST14* [23], as well as in four additional patients from three families [18,24]. These disorders were defined as subtypes of EDS, based on the International Classification of the EDSs [3]. Clinical features are highly characteristic, comprising multisystem congenital malformations such as craniofacial features (e.g., large fontanelle, hypertelorism, short and downslanting palpebral fissures, blue sclerae, short nose with hypoplastic columella, low-set and rotated ears, high palate, long philtrum, thin upper lip vermilion, small mouth and micro-retrognathia), multiple congenital contractures (e.g., adduction–flexion contractures of thumbs and talipes equinovarus), and visceral and ocular malformations. Features also include progressive connective tissue fragility-related manifestations, such as skin hyperextensibility, bruisability, and fragility with atrophic scars; recurrent dislocations; progressive talipes or spinal deformities; pneumothorax or pneumohemothorax; large subcutaneous hematomas; and/or diverticular perforation (Figure 1) [1,2]. Major diagnostic criteria of the disorder are as follows: 1) congenital multiple contractures, characteristically adduction–flexion contractures and/or talipes equinovarus (clubfoot); 2) characteristic craniofacial features, which are evident at birth or in early infancy; 3) characteristic cutaneous features including hyperextensibility, bruisability and fragility with atrophic scars, as well as increased palmer wrinkles [3]. Minor criteria as follows: 1) recurrent/chronic dislocations, 2) pectus deformities (e.g., flat or excavated), 3) spinal deformities (e.g., scoliosis or kyphoscoliosis), 4) peculiar fingers (e.g., tapered, slender, or cylindrical), 5) progressive talipes deformities (e.g., valgus, planus, or cavum), 6) large subcutaneous hematomas, 7) chronic constipation, 8) colonic diverticula, 9) pneumothorax/pneumohemothorax, 10) nephrolithiasis/cystolithiasis, 11) hydronephrosis, 12) cryptorchidism in males, 13) strabismus, 14) refractive errors (e.g., myopia or astigmatism) and/or 15) glaucoma/elevated intraocular pressure [3]. 

In this review, we describe the comprehensive pathophysiological findings of mcEDS, as demonstrated in previous studies including our recent reports.

## 2. Molecular Findings

**Figure 2:**
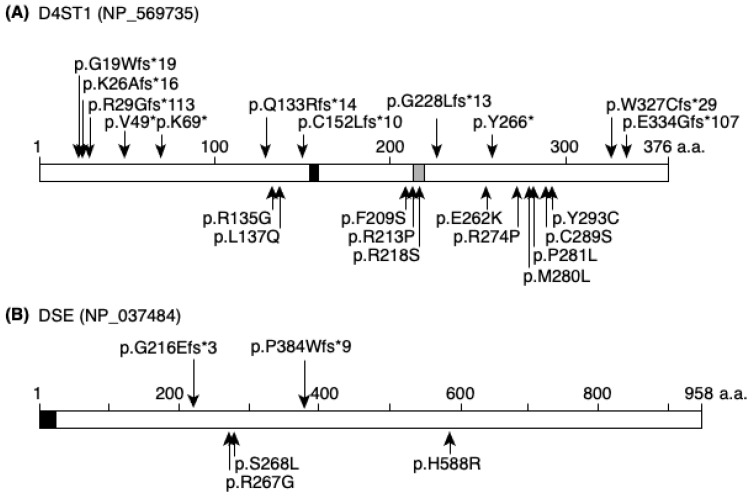
Published pathogenic protein changes of D4ST1 and DSE in mcEDS. (**A**) Previously published truncating and non-truncating protein alterations in D4ST1 are shown in upper and lower panels, respectively. Black box indicates 5′-phosphosulfate binding site and gray box indicates 3′-phosphate binding site. (**B**) Previously published truncating and non-truncating protein alterations in DSE are shown in upper and lower panels, respectively. Black box indicates the signal peptide.

Pathogenic variants have been detected throughout *CHST14* (NM_130468.4): 11 missense variants, five frameshift variants, and three nonsense variants in patients with mcEDS [4,6,7,14,15,16,17,18,19,22] (Figure 2). The p.(Pro281Leu) variant is most common (*n =* 10 families), followed by p.(Try293Cys) (*n =* 4), p.(Val49*) (*n =* 3), p.(Arg213Pro), and p.(Phe209Ser) (*n =* 2); p.(Arg29Glyfs*113), p.(Lys69*), p.(Gln113Argfs*14), p.(Arg135Gly), p.(Leu137Gln), p.(Cys152Leufs*10), p.(Arg218Ser), p.(Gly228Leufs*13), p.(Glu262Lys), p.(Tyr266*), p.(Arg274Pro), p.(Met280Leu), p.(Cys289Ser), p.(Trp327Cysfs*29), and p.(Glu334Glyfs*107) variants are particularly uncommon (*n =* 1 for all). Furthermore, p.(Gly19Trpfs*19) and p.(Lys26Alafs*16) variants have been detected in patients with features similar to those of mcEDS, among patients with hereditary connective tissue disorders and skeletal dysplasia, respectively (Figure 2A) [25,26]. Three missense variants have been detected in *DSE* (NM_013352.4): p.(Arg267Gly), p.(Ser268Leu), and p.(His588Arg); and one frameshift variant, p.(Pro384Trpfs*9), has also been detected (Figure 2B) [18,23,24]. Another frameshift variant, p.(Gly216Glufs*3), was detected in a patient with features similar to those of mcEDS, among patients with chondrodysplasia who exhibited multiple dislocations (Figure 2B) [27].

No apparent genotype-phenotype correlations have been reported in patients with mcEDS-*CHST14*. Phenotypes of patients with mcEDS-*DSE* seem to be milder than those of patients with mcEDS-*CHST14* [18,24].

## 3. Glycobiological Findings

**Figure 3:**
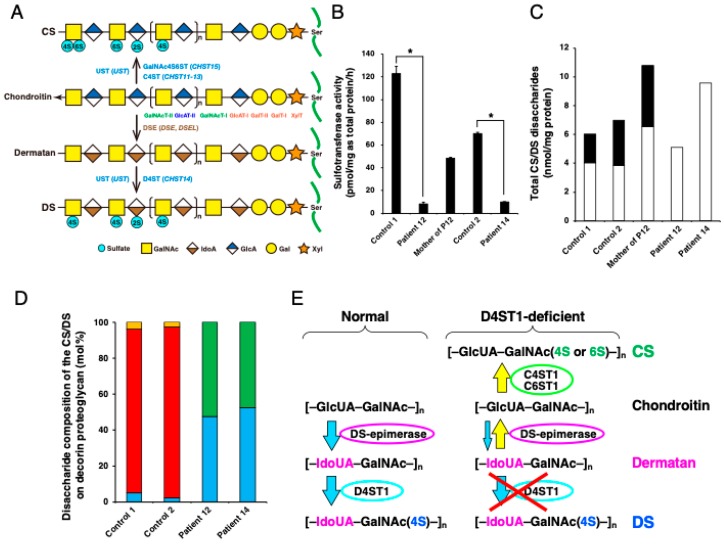
Biosynthesis of CS/DS and glycobiological analysis in mcEDS-*CHST14*. (**A**) Biosynthetic assembly of CS and DS chains by glycosyltransferases, epimerases, and sulfotransferases. It starts with the biosynthesis of a tetrasaccharide linker region, glucuronic acid-β1,3-galactose-β1,3-galactose-β1,4-xylose-β1-*O*-(GlcUA-Gal-Gal-Xyl-), onto serine residues of specific core proteins of PGs by β-xylosyltransferase (XylT), GalT-I, GalT-II and β1,3-glucuronosyltransferase-I (GlcAT-I), respectively. Subsequently, a repetitive disaccharide region [*N*-acetyl-d-galactosamine(GalNAc)-GlcUA)]_n_ of chondroitin is elongated by the actions of *N*-acetyl-d-galactosaminyltransferase-I (GalNAcT-I), *N*-acetyl-d-galactosaminyltransferase-II (GalNAcT-II) and glucuronyltransferase-II (GlcAT-II), which are encoded by CS *N*-acetylgalactosaminyltransferase-1 and -2, chondroitin synthase-1, -2, and -3 and chondroitin polymerizing factor genes. Chondroitin chains are matured to CS through modifications by chondroitin 4-*O*-sulfotransferase (C4ST), chondroitin 6-*O*-sulfotransferase (C6ST) and uronyl 2-*O*-sulfotransferase (UST). A disaccharide-repeating region of dermatan is synthesized through epimerization of a carboxyl group at C5 from GlcUA to IdoUA by DSE. A mature DS chain is synthesized through modification with sulfation by D4ST1 and UST. (**B**) Sulfotransferase activity toward dermatan in fibroblast cultures of skin from two patients with mcEDS-*CHST14* (patient 12 with heterozygous variants Pro281Leu/Tyr293Cys; patient 14 with a homozygous variant Pro281Leu), the mother of patient 12, and a sex- and age-matched healthy volunteer [6]. * *p* < 0.0001 by two-tailed unpaired *t*-test. (**C**) Total amounts of CS and DS derived from fibroblast cultures of skin [6]. Total disaccharide contents of CS (white box) and DS (black box) were calculated based on the peak area in the chromatograms of the digests with chondroitinase AC and chondroitinase B, respectively. (**D**) Proportion of the disaccharide units in the CS-DS hybrid chain in decorin-PGs secreted by the fibroblasts [6]. Cyan, green, red and orange boxes are GlcUA-GalNAc(4S), GlcUA-GalNAc(6S), IdoUA-GalNAc(4S), and IdoUA(2S)-GalNAc(4S), respectively. Abbreviations of 2S, 4S and 6S indicate 2-*O*-, 4-*O*- and 6-*O*-sulfate, respectively. (**E**) Schematic diagram of the biochemical mechanism in the replacement of DS by CS in mcEDS-*CHST14*. Defect in D4ST1 enables a back-epimerization reaction that converts IdoUA back to GlcUA to form chondroitin by DSE, followed by the 4-*O*-sulfation and/or 6-*O*-sulfation of GalNAc residues in chondroitin by C4ST1 and C6ST1, respectively. (B–D, reproduced from Miyake et al. *Hum. Mutat.* **2010**, *31*, 1233–1239, with permission from Wiley-Liss, Inc.).

Normal, biosynthetic pathways of chondroitin sulfate (CS) and dermatan sulfate (DS) are shown in Figure 3A. Reduced activity of D4ST1 in fibroblast cultures of skin from a patient with mcEDS-*CHST14* caused by compound heterozygous p.(Pro281Leu)/(Tyr293Cys) substitutions in *CHST14*, as well as in fibroblasts from a patient with mcEDS-*CHST14* caused by a homozygous p.(Pro281Leu) substitution in *CHST14*, showed a marked reduction in D4ST1 activity (Figure 3B); this change in activity resulted in a negligible amount of DS and an excessive amount of CS (Figure 3C) [6]. 

Decorin, which consists of a core protein and a single glycosaminoglycan (GAG) chain, is a major DS-proteoglycan (PG) that plays an important role in the assembly of collagen fibrils in the skin [28]; it also plays roles in the pathophysiology of mcEDS-*CHST14* [1,2,3,4,6,7]. GAG chains of decorin-PG from skin fibroblasts of a patient with p.(Pro281Leu)/(Tyr293Cys) substitutions, as well as from skin fibroblasts of a patient with a homozygous p.(Pro281Leu) substitution, contained only CS and no DS; in contrast, GAG chains of decorin-PG from skin fibroblasts of healthy controls contained mainly DS (Figure 3D) [6,18]. 4-*O*-Sulfation in CS and DS chains functions as an inhibitor of DSE [29]. Thus, impaired 4-*O*-sulfation inhibition due to D4ST1 deficiency enables back-epimerization from L-iduronic acid (IdoUA) to D-glucuronic acid (GlcUA) (Figure 3E) [4,6,7]. In our laboratory, we have established a urinary disaccharide analysis of CS/DS chains through an anion-exchange chromatography after treatment with DS-specific degrading enzymes; this analysis method showed that no DS was present in the urine of eight patients with mcEDS-*CHST14* [30]. This result suggested a systemic depletion of DS in patients with mcEDS-*CHST14*; thus we presume that our urinary disaccharide analysis method can be implemented to allow a non-invasive screening for mcEDS-*CHST14* [30].

Regarding patients with mcEDS-*DSE*, reduced activity of DSE in fibroblast cultures of skin from a patient with a homozygous p.(Ser268Leu) substitution resulted in marked reduction of DS disaccharides, compared with healthy controls [23]. The total amount of CS in the cell fraction from affected skin fibroblasts was increased by approximately 1.5-fold, which might reflect increased synthesis and/or reduced conversion of CS chains [23]. A minor fraction of DS from decorin-PG was present in skin fibroblasts from a patient with a homozygous p.(Ser268Leu) substitution [23]; this suggested residual DSE activity or compensation by DSE2, which might be related to milder phenotypes in patients with mcEDS-*DSE* than in patients with mcEDS-*CHST14* [18].

## 4. Pathological Findings

**Figure 4:**
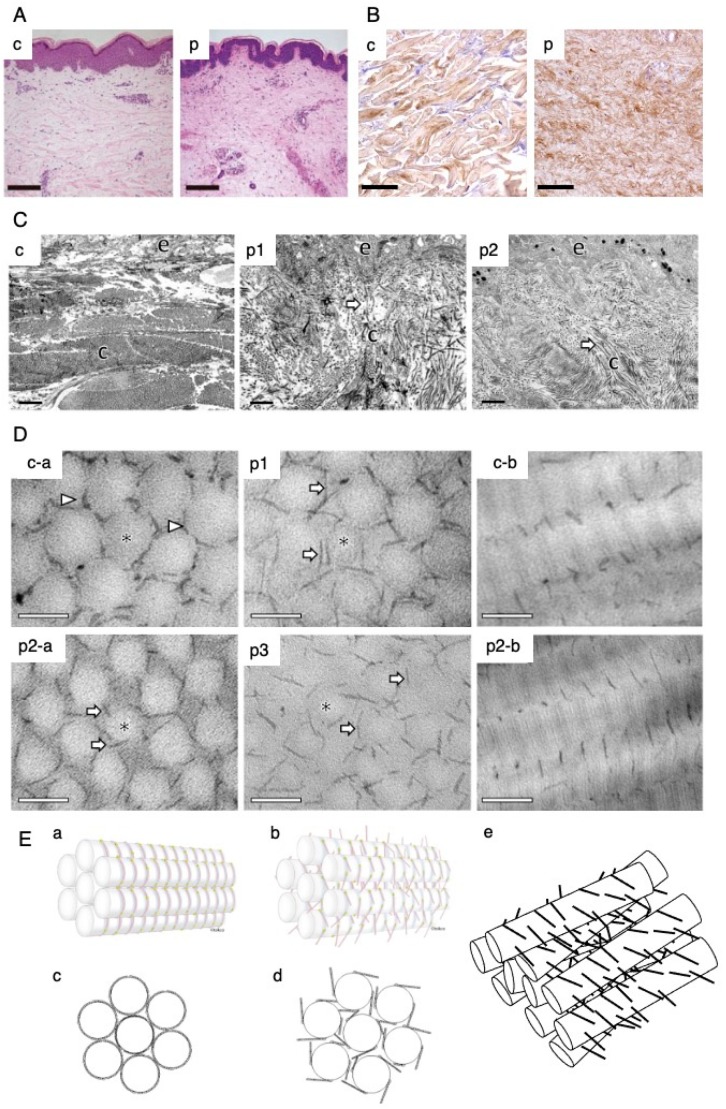
Skin pathology of mcEDS-*CHST14*. (**A**) Light microscopy (hematoxylin and eosin staining). In the skin specimen from a patient with heterozygous variants Pro281Leu/ Cys289Ser (panel p), fine collagen fibers are present predominantly in the reticular to papillary dermis with marked reduction of thick collagen bundles; thick collagen bundles are observed in a skin specimen from a healthy control volunteer (panel c) [6]. (**B**) Immunohistochemical staining for decorin core protein. Decorin core protein is present on collagen fibers in thick bundles in a skin specimen from a healthy control volunteer (panel c), but on thin and filamentous collagen fibers without clear boundaries in a skin specimen from a patient with heterozygous variants Pro281Leu/ Cys289Ser (panel p) [31] (**C**) Transmission electron microscopy. Collagen fibrils are regularly and tightly assembled in a skin specimen from a healthy control volunteer (panel c), but are dispersed in the papillary to reticular dermis in skin specimens from a patient with heterozygous variants Pro281Leu/ Tyr293Cys (panel p1) and a patient with a novel homozygous variant (panel p2) [31]. (**D**) Transmission electron microscopy-based cupromeronic blue staining. GAG chains are curved and maintain close contact with attached collagen fibrils in the skin specimens from a healthy control volunteer (panels c-a, c-b); conversely, they are linear and stretch from the outer surface of collagen fibrils to adjacent fibrils in skin specimens from a patient with heterozygous variants Pro281Leu/ Tyr293Cys (panel p1), a patient with a novel homozygous variant (panels p2a and p2b) and another patient with heterozygous variants Pro281Leu/ Tyr293Cys (panel p3) [31]. (**E**) Schematic representations of collagen fibrils and GAG chains. Decorin core protein binds to D bands of collagen fibrils both in normal skin and in affected skin. GAG chains composed of DS adhere to collagen fibrils along D bands, beginning from the core protein (panels a and c), whereas GAG chains composed of CS extend linearly and perpendicularly to collagen fibrils from the core protein (panels b and d) [31]. Putative spatial disorganization of collagen fibril networks in the skin of patients (panel e) (A, reproduced from Miyake et al. *Hum. Mutat.* **2010**, *31*, 1233–1239, with permission from Wiley-Liss, Inc.; B–E, reproduced from Hirose et al. *Biochim. Biophys. Acta Gen. Subj.* **2019**, *1863*, 623–631, with permission from Elsevier, Inc.).

The pathology of mcEDS-*CHST14*, as the simplest model for complete depletion of DS, has been extensively investigated using affected skin specimens. Light microscopy of skin specimens (hematoxylin and eosin staining) from patients with compound heterozygous p.(Pro281Leu)/(Cys289Ser) or p.(Pro281Leu)/(Tyr293Cys) substitutions showed that fine collagen fibers were predominantly present in the reticular to papillary dermis; marked reduction of normally thick collagen bundles were also observed (Figure 4A) [6]. Immunohistochemistry staining of decorin core protein in skin specimens from patients with compound heterozygous p.(Pro281Leu)/(CYs289Ser) or p.(Pro281Leu)/(Tyr293Cys) substitutions showed that decorin core protein was present on collagen fibers that were thin and filamentous without clear boundaries; in contrast, skin specimens from healthy controls showed decorin core protein on collagen fibers that were thick bundles with clear boundaries [31] (Figure 4B). Transmission electron microscopy of skin specimens from five patients with compound heterozygous p.(Pro281Leu)/(Cys289Ser), p.(Pro281Leu)/(Tyr293Cys), or p.(Phe209Ser)/(Pro281Leu) substitutions showed that collagen fibrils were dispersed in the papillary to reticular dermis, whereas skin specimens from healthy controls exhibited collagen fibrils that were regularly and tightly assembled (Figure 4C) [6,31]. Transmission electron microscopy-based cupromeronic blue staining to visualize GAG chains on affected skin samples showed that GAG chains were linear, stretching from the outer surface of collagen fibrils to adjacent fibrils, whereas skin samples from healthy controls exhibited curved GAG chains that maintained close contact with attached collagen fibrils (Figure 4D) [31]. This structural alteration of GAG chains of decorin is presumably related to the biochemical alteration from DS to CS: the structure of DS-GAG chains is flexible because L-IdoUA residues in DS can easily adopt any of the nearly equi-energetic ^1^C_4_, ^2^S_0_, and ^4^C_1_ conformations, whereas the structure of CS-GAG chains is rigid because D-GlcUA in CS only adopts the ^4^C_1_ conformation [6,32,33]. 

Furthermore, focused ion beam scanning electron microscopy using cupromeronic blue staining uncovered the structure of collagen fibrils in association with GAG chains (likely comprising decorin): GAG chains form a ring mesh-like structure with each ring surrounding a collagen fibril at its D band, fusing with adjacent rings to form a planar network [34]. Abnormally stretching CS-GAG chains of decorin in the affected skin would disrupt the ring-mesh structure of collagen fibrils (Figure 4E), which could result in substantial fragility.

## 5. Animal Model-Based Findings

**Figure 5:**
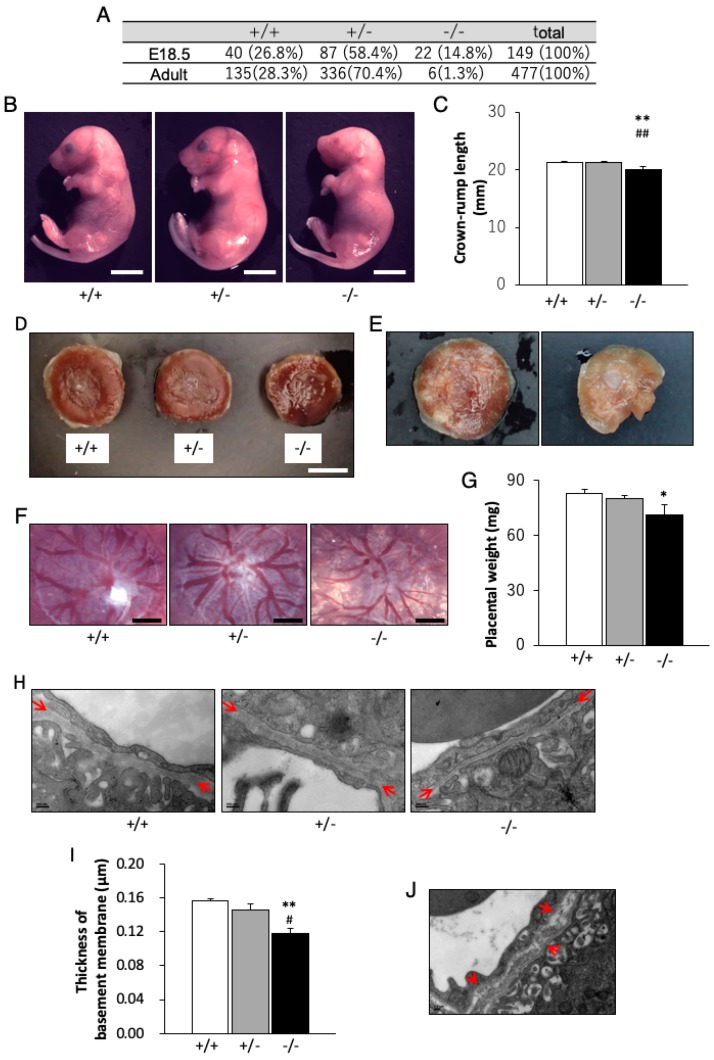
Mouse model for mcEDS-*CHST14*. (**A**) Numbers (percentages) of each type at embryonic (E18.5) and postnatal (adult) ages. Markedly greater numbers of homozygous fetuses are observed, compared with homozygous adults. (**B**) General appearances of wild-type (*Chst14*^+/+^), heterozygous (*Chst14*^+/−^) and homozygous (*Chst14*^−/−^) fetuses [37]. (**C**) Crown-rump length of each type of fetus (bar: 5 mm). Homozygous mice demonstrate significantly shorter crown-rump length, compared with wild-type or heterozygous mice [37]. (**D**) Appearance of each type of placenta (bar: 5 mm) [37]. (**E**) Homozygous placentas exhibit appearances indicative of hypoxia (left) and necrosis (right) [37]. (**F**) Microphotographs of the chorionic plate side of the placentas (bar: 1 mm). Homozygous placentas demonstrate smaller vascular diameters, compared with wild-type or heterozygous placentas [37]. (**G**) Weight of each type of placenta (mean + standard error of the mean). (**H**) Transmission electron microscopy of capillary basement membrane in the labyrinth zone of each type of placenta (bar: 200 nm). Arrows indicate capillary basement membrane [37]. (**I**) Homozygous placentas show a significantly thinner capillary basement membrane, compared with wild-type or heterozygous placentas [37]. (**J**) Homozygous placentas show structural abnormalities (arrows) in capillary basement membrane (bar: 0.2 μm) [37]. * *p* < 0.05, ** *p* < 0.01, compared with wild-type; # *p* < 0.05, ## *p* < 0.01, compared with heterozygous; one-way analysis of variance (ANOVA) followed by the Tukey–Kramer post hoc test. (A–J, reproduced from Yoshizawa et al. *Glycobiology* **2018**, *28*, 80–89, with permission from Oxford University Press, Inc.).

Knockout (*Chst14*^−/−^) mice were generated through homologous recombination that targeted the only coding exon 1 (i.e., exon 1) of *Chst14* [35,36]. F2 mice showed reduced weight and/or length and reduced bone volume/thickness/density of their lumbar vertebrae [35]. *Chst14*^−/−^ mice showed reduced neurogenesis and diminished proliferation of neural stem cells, accompanied by increased expression of glutamate aspartate transporter and epidermal growth factor, compared with findings in both wild-type (*Chst14*^+/+^) mice and other knockout (*Chst11*^−/−^) mice, a model for chondroitin 4-*O*-sulfotransferase 1 (C4ST1) deficiency [36]. *Chst14*^−/−^ mice also had a smaller body mass (Figure 5A, B), reduced fertility, a kinked tail, and increased skin fragility compared with their wild-type (*Chst14*^+/+^) littermates; however, brain weight and gross anatomy were not affected [36,37]. Schwann cells from *Chst14*^−/−^ mice formed longer processes in vitro and exhibited greater proliferation than those from *Chst14*^+/+^ mice. Functional recovery and axonal regrowth in *Chst14*^−/−^ mice were initially accelerated after femoral nerve transection and suture; after 3 months, these characteristics were similar to those in *Chst14*^+/+^ littermates. These findings suggested that DS, synthesized by Chst14/D4st1, might be of limited importance for neural development; moreover, it might contribute to the regeneration-restricting environment in the adult mammalian nervous system [38]. Only a few adult *Chst14*^−/−^ mice were generated because of perinatal lethality in most of the homozygous mice; these adult *Chst14*^−/−^ mice showed significantly shorter crown-rump length, compared with wild-type or heterozygous mice (Figure 5A–C) [37,38]. The placentas of *Chst14*^−/−^ fetuses showed a reduced weight, alterations in the vascular structure and ischemic and/or necrotic-like changes (Figure 5D–G). Transmission electron microscopy of homozygous placentas demonstrated an abnormal capillary basement membrane structure in the placental villus, compared with wild-type or heterozygous placentas (Figure 5H–J). These findings showed that DS was essential for placental vascular development and perinatal fetal survival. In addition, DS was suggested to be related to the structure and/or function of capillary basement membranes, which constitutes an extracellular matrix [37].

C4st1, encoded by *Chst11*, transfers a sulfate group from 3′-phosphoadenosine 5′-phosphosulfates to the C4 position of GalNAc residues in chondroitin (Figure 3A). *Chst11*-mutant mice died within 6 hours of birth due to respiratory failure, severe dwarfism and chondrodysplasia (i.e., abnormalities in the cartilage growth plate and chondrocyte columns) [39]. Furthermore, marked reductions in the content and 4-*O*-sulfation of CS, as well as the downregulation of bone morphogenetic protein signaling and upregulation of transforming growth factor-β, have also been observed in *Chst11*-mutant mice. These findings suggest that Chst11/C4st1 and the 4-*O*-sulfation of CS chains are essential for early development and for bone morphogenetic protein and transforming growth factor-β in signaling pathways in cartilage. When considered in the context of *Chst14*^−/−^ mouse phenotypes, the above findings suggest that 4-*O*-sulfation is required for the maturation of both CS and DS; moreover, CS and DS exert distinct functions in the development of cartilage and skin, respectively. However, the DS content in *Chst11*-mutant mice remains to be elucidated [39]. 

Biosynthesis of DS requires D4st1, dermatan sulfate epimerase 1 (DS-epi1) and dermatan sulfate epimerase 2 (DS-epi2), which are encoded by *Chst14*, *Dse* and *Dsel*, respectively [40,41,42,43]. Furthermore, D4ST1 interacts directly with DS-epi1, but not with DS-epi2, to form a hetero-complex that is required for the formation of IdoUA blocks in DS chains [44]. *DS-epi1*^−/−^ mice showed greater skin fragility compared with wild-type littermates, due to altered collagen fibril morphology [45]; this phenotype was similar to that of *Chst14*^−/−^ mice [38]. The numbers of IdoUA blocks were dramatically reduced in DS side chains of decorin-PG and biglycan-PG from the skin of *DS-epi1*^−/−^ mice [45], which suggests that DS-epi1 mainly synthesizes IdoUA blocks in vivo. *DS-epi1*^−/−^ embryos and newborn mice showed kinked tails, which is a common feature in *Chst14*^−/−^ mice [38]; the *DS-epi1*^−/−^ embryos and newborn mice also showed significantly thicker epidermal layers through histological staining, compared with heterozygous or wild-type littermates [45,46]. Immunohistochemical staining of epidermal layers in *DS-epi1*^−/−^ newborns showed increased expression of keratin 5 in the basal layer and keratin 1 in the spinous layer [46]. Furthermore, a small portion of *DS-epi1*^−/−^ embryos showed an abdominal wall defect with herniated intestines, exencephaly and spina bifida. Defective collagen structure in the dermis and imbalanced keratocyte maturation were presumed to cause developmental defects in *DS-epi1*^−/−^ mice [46]. These observations indicate that *DS-epi1*^−/−^ mice may constitute a useful model of mcEDS-*DSE*. *DS-epi2*^−/−^ mice displayed no significant defects and DS-epi1 compensated for the absence of DS-epi2 in most tissue, which indicated that DS-epi1 is the major contributor of epimerase activity [47]. DS-epi2 exhibits higher expression than DS-epi1 in developing the mouse brain [48]. Although CS/DS chains in the brains of *DS-epi2*^−/−^ newborn mice demonstrated a 38% reduction in IdoUA content, compared with wild-type littermates, the brains of adult knockout mice showed normal extracellular matrix features [47]. *DS-epi1*^−/−^/*DS-epi2*^−/−^ mice experienced perinatal death with variable phenotypes at late embryological stages and birth; these phenotypes included umbilical hernia, exencephaly, and a kinked tail, as well as complete loss of IdoUA residues in CS/DS chains [49]. However, a minority of embryos exhibited normal lung, bone, and cartilage features. 

These findings indicate that DS, DS-PGs, DS-epi1 and/or DS-epi2 are important in early embryonic development and perinatal survival [49].

## 6. Ongoing Projects and Future Perspectives

Our group established a multicenter collaboration network to promote comprehensive basic research on mcEDS. In this network, genetic testing is provided as part of routine medical care covered by national health insurance, using a custom next generation sequencing-based panel that includes all EDS-related genes and genes for hereditary connective tissue disorders [50]. Whole exome sequencing is performed to identify other potential causative genes for mcEDS in patients with similar features who do not exhibit pathogenic variants in *CHST14* or *DSE*. Biochemical abnormalities of the extracellular matrix are investigated (e.g., various types of collagen and DS-PGs, including decorin and biglycan), using patient skin fibroblasts. Furthermore, mass spectrometry is performed to identify appropriate serum/plasma biomarker(s) that may be useful in the diagnosis or surveillance of disease progression. A subsequent pathological approach includes an elucidation of the structural alterations of collagen fibril networks and GAG chains of decorin in skin specimens from patients with mcEDS-*DSE*. Determination of the crystal structure of D4ST1 would be useful for understanding the effects of common missense variants (e.g., p.(Pro281Leu)) in *CHST14*. A technical breakthrough in the efficient generation of knockout mice for *Chst14* (e.g., CRISPR/cas9) is needed to continue mouse-based phenotypic and pathophysiological investigations. Considering the differences in the phenotypes between patients with mcEDS and *Chst14*^−/−^ or *DS-epi1*^−/−^ mice, experiments using other models that could reflect phenotypic and pathophysiological abnormalities in patients with mcEDS, such as induced pluripotent stem cells, would be particularly useful. All of these approaches will be valuable for further elucidating the critical roles of DS and DS-PGs, including decorin and biglycan, in the multisystem development and maintenance of connective tissues; in addition, they provide fundamental evidence for future etiology-based therapies, such as adeno-associated virus-based gene therapy.
